# Editorial: Investigating tumor immunotherapy responses in lung cancer using deep learning

**DOI:** 10.3389/fimmu.2024.1529949

**Published:** 2024-12-03

**Authors:** Shuang Qin, Haoxiang Zhang, Chao Liu, Ming Yi

**Affiliations:** ^1^ Department of Radiation Oncology, Hubei Cancer Hospital, Tongji Medical College, Huazhong University of Science and Technology, Wuhan, China; ^2^ Department of Hepatopancreatobiliary Surgery, Shengli Clinical Medical College of Fujian Medical University, Fuzhou University Affiliated Provincial Hospital, Fuzhou, China; ^3^ Department of Radiation Oncology, Shandong Cancer Hospital and Institute, Shandong First Medical University and Shandong Academy of Medical Sciences, Jinan, China; ^4^ Department of Breast Center, The First Affiliated Hospital, College of Medicine, Zhejiang University, Hangzhou, China

**Keywords:** tumor microenvironment, cancer immunotherapy, deep learning, artificial intelligence, lung cancer

**Figure 1:**
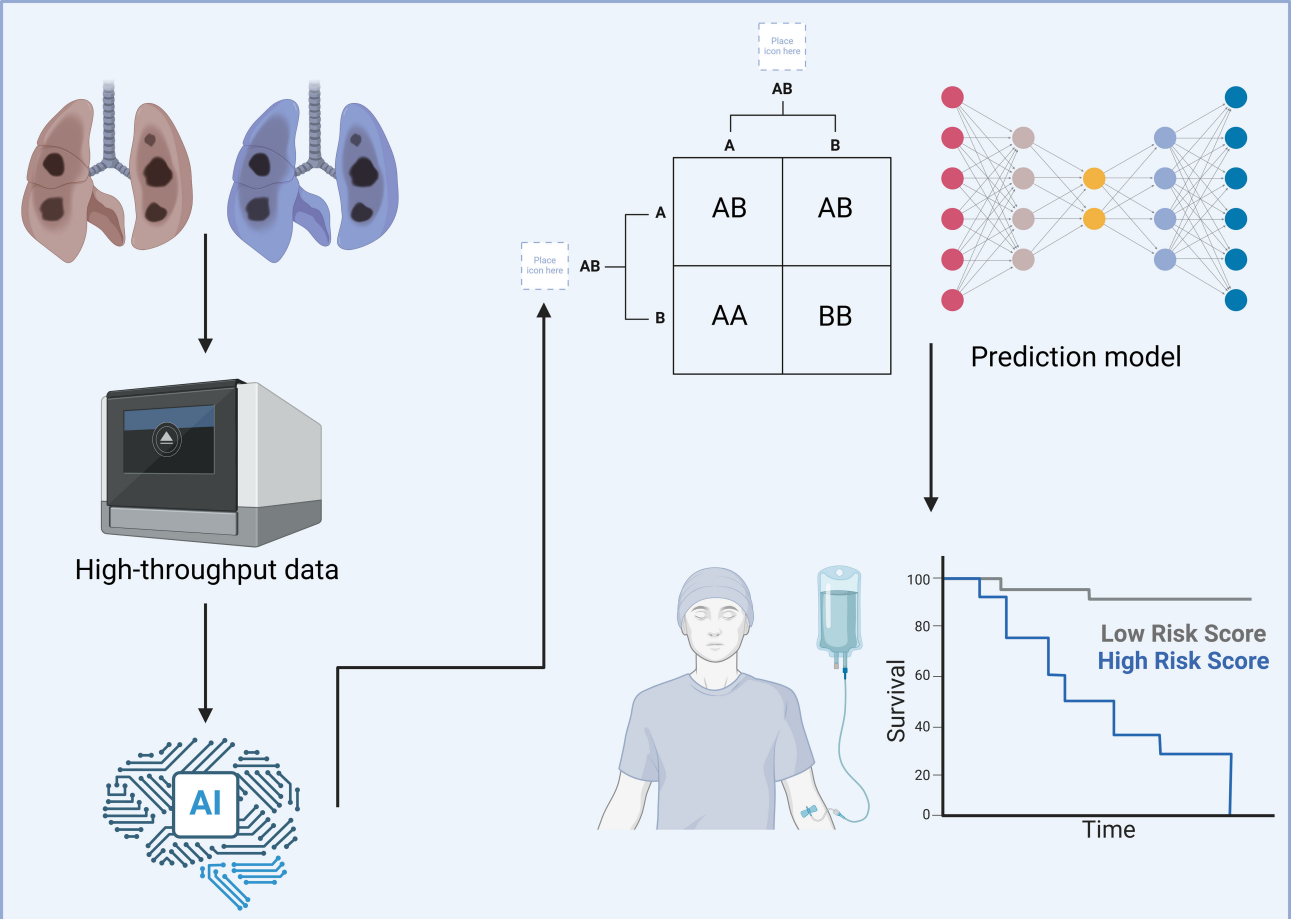
Using deep learning to explore the tumor microenvironment of lung cancer. This figure illustrates a detailed workflow utilizing deep learning technologies to comprehensively analyze the TME in lung cancer cases. The aim is to enhance patient stratification and personalize therapeutic approaches more effectively. High-throughput data are gathered from lung cancer tissues, encompassing diverse molecular characteristics including, but not limited to, genetic mutations, immune cell composition, and biomolecular interactions. This data is processed using an advanced AI-based framework designed to handle complex, multidimensional datasets with efficiency. The AI system employs sophisticated algorithms to categorize this complex data into distinct molecular and cellular profiles. It identifies specific patterns and associations, such as particular genetic mutations, and correlates these with various risk profiles observed in lung cancer progression. These risk profiles are crucial for understanding the aggressiveness and potential treatment responses in different patient groups. Following the data categorization, they are input into a predictive model that employs a deep neural network architecture. This model integrates TME characteristics with clinical outcomes, specifically focusing on survival rates. It effectively stratifies patients into high- and low-risk categories based on their molecular and cellular markers, as visually represented in the survival curves. This strategic classification facilitates the development of tailored treatment plans, enabling oncologists to target therapies that are most likely to be effective for specific patient profiles. Created with BioRender.com.

The treatment paradigm for lung cancer has evolved substantially over the past few decades (1). Initially centered around traditional modalities such as radiotherapy and chemotherapy, the field has now shifted toward more sophisticated interventions, including targeted therapies and immunotherapy (2). While these innovations have indeed improved outcomes and quality of life for many patients, a significant portion of individuals still do not experience substantial benefit from these advanced therapies (3). In response, researchers have increasingly turned to multi-omics technologies to better understand the complex biology underlying lung cancer and its interactions within the immune microenvironment (4). These high-throughput data, encompassing genomic, transcriptomic, proteomic, and metabolomic layers, offer rich information on potential therapeutic targets and prognostic markers that could redefine lung cancer treatment (**Figure 1**) (5, 6). However, the sheer scale and complexity of these datasets pose a critical interpretative challenge for clinicians and scientists alike.

Enter deep learning, an advanced form of artificial intelligence (AI) that has proven instrumental in decoding large, complex datasets with unprecedented precision (7). Unlike traditional data analysis techniques, deep learning can parse intricate biological relationships and uncover novel oncogenic pathways that may otherwise remain obscured (8). Through these capabilities, deep learning provides an invaluable lens through which researchers can gain insight into the dynamic processes driving lung cancer progression. It enables the in-depth analysis of complex phenomena such as cell signaling interactions, immune responses, and metabolic reprogramming within the tumor microenvironment (9). As deep learning continues to advance, it holds the promise of not only improving our understanding of malignant biological behaviors but also driving the development of precision oncology strategies tailored to individual patients’ unique disease profiles (10).

This Research Topic presents seven articles that investigate the role of deep learning in studying lung cancer, with a particular focus on the tumor immune microenvironment. These studies explore different ways deep learning is being applied to analyze the immune landscape, offering valuable perspectives on how AI can enhance our ability to predict therapeutic responses and identify new therapeutic targets.

One such study, conducted by Zheng et al., examines the impact of STK11 mutations on patient outcomes in non-small cell lung cancer (NSCLC). In an analysis of 188 NSCLC patients, the researchers found that high STK11 expression correlates with improved progression-free and overall survival, an observation further substantiated by data from the TCGA cohort. However, when mutated, STK11 is associated with poorer outcomes in both lung squamous cell carcinoma and adenocarcinoma subtypes. To investigate these findings further, the team conducted bioinformatics analyses that revealed seven immune-related genes (CALCA, BMP6, S100P, THPO, CGA, PCSK1, and MUC5AC) that were overexpressed in STK11-mutated tumors. This overexpression suggests that STK11 mutation may drive specific changes in immune gene expression, which in turn can affect NSCLC prognosis. These data underscore the complex role of STK11 in lung cancer and demonstrate its potential as a target for more personalized therapeutic approaches.

Another important study in this Research Topic, led by Liu et al., explores the development of a novel AI-based immunoscore called the patho-immunoscore to predict outcomes in advanced non-squamous NSCLC patients undergoing chemoimmunotherapy. Using over 1,300 whole-slide images from the TCGA-LUAD dataset, the researchers built a model that demonstrated robust predictive performance, which was further validated across independent study cohorts, including CPTAC-LUAD and ORIENT-11. A high patho-immunoscore was associated with significantly improved progression-free survival in patients receiving chemoimmunotherapy, highlighting the potential of AI-driven immunoscoring as a powerful prognostic tool. Importantly, these results suggest that the patho-immunoscore may be a broadly applicable biomarker not only for NSCLC but also for other cancer types where immunotherapy plays a crucial role.

The need for accurate biomarkers to predict responses to immunotherapy in NSCLC is further explored in an insightful review by (Zheng et al.). They argue that conventional imaging approaches, which primarily capture macroscopic tumor changes, may fall short in meeting the precision required by modern cancer diagnosis and treatment. In contrast, CT and PET/CT radiomics can reveal molecular-level features, such as PD-1/PD-L1 expression and tumor mutation burden, that hold potential as indicators of immunotherapy efficacy and patient prognosis. By integrating radiomics with machine learning and AI, the researchers propose a novel diagnostic framework capable of assessing not only the therapeutic response but also the likelihood of immune-related side effects. This review positions radiomics as a promising non-invasive tool for predicting immunotherapy benefits in NSCLC, with the potential to facilitate more personalized treatment plans.

Exploring novel therapeutic targets, Xu et al. conducted a comprehensive review of the STING pathway and its potential role in enhancing cancer immunotherapy, particularly for patients with low response rates to anti-PD-1/PD-L1 therapies. STING (stimulator of interferon genes) plays a critical role in processes such as antigen presentation and the DNA damage response, which are essential for effective anti-tumor immunity. The researchers reviewed various STING agonists, including cyclic dinucleotides (CDNs) and non-CDN-based agents, and summarized advances in delivery systems, such as nanocarriers and antibody-drug conjugates, that improve STING agonist targeting and safety profiles. By enhancing STING activation, these therapies show potential for combination strategies that could address common resistance mechanisms in immunotherapy.

Real-world challenges in immunotherapy are exemplified in a case report by Xia et al., detailing a patient with advanced lung cancer who experienced three consecutive severe immune-related adverse events (irAEs) after treatment with sintilimab and chemotherapy. This report underscores the need for a better understanding of irAEs, which are complex immune responses that can severely impact patient quality of life and treatment adherence. The authors also reviewed current research on biomarkers for early irAE prediction, with the hope of improving management strategies for irAEs associated with immune checkpoint inhibitors.

Lastly, Shi et al. present cases of extensive-stage small-cell lung cancer patients who developed paraneoplastic neurological syndrome (PNS) following treatment with ICIs and chemotherapy. The study details neurological symptoms and positive paraneoplastic antibody tests, underscoring the need for clinicians to consider PNS as a possible complication of ICI therapy. This report highlights the importance of timely diagnosis and tailored management of ICI-induced neurological complications, advocating for standardized testing protocols to enhance patient care.

Collectively, the studies in this Research Topic underscore the transformative potential of deep learning in lung cancer research, particularly as it relates to the tumor immune microenvironment. By leveraging deep learning algorithms, researchers are gaining new insights into lung cancer biology, refining our understanding of immunotherapy responses, and identifying novel therapeutic targets. As deep learning technologies continue to evolve, they will undoubtedly play a vital role in advancing the precision of lung cancer treatment and expanding the promise of personalized oncology.
